# A quasi-experimental study to explore the association between nutritional education within the Holiday Activities and Food programme and related outcomes for children

**DOI:** 10.1017/S1368980026102225

**Published:** 2026-02-27

**Authors:** Emily K. Hope, Paul B. Stretesky, Margaret Anne Defeyter

**Affiliations:** 1 Northumbria University, UK; 2 University of Lincoln, UK

**Keywords:** Nutritional education, Holiday provision, Holiday clubs, Food education, Food literacy, Food insecurity, School policy

## Abstract

**Objective:**

Across school and community-based contexts, nutritional education interventions are often associated with improvements in a range of food-related and health-related outcomes. The aim of this study was to investigate whether the nutritional education component of the Holiday Activities and Food (HAF) programme in England was similarly associated with changes in these outcomes for children who attend.

**Design:**

A quasi-experimental, mixed-factorial 3 (School) × 3 (Group) × 2 (Time) design was employed. Outcome variables were liking and frequency of trying new foods, perceived cooking competence and health-related quality of life.

**Setting:**

Pre-post data were collected at three primary schools in one local authority in the North East of England at two time points (before and after the summer holidays).

**Participants:**

A non-probability, purposive sample of 169 children (mean age = 9·4 years, sd = 0·54) self-selected into groups of children who did not attend HAF over the summer holidays (No HAF; *n* 123), attended their school-based HAF club (HAF; *n* 29) or attended their school-based HAF club alongside a bespoke nutritional education programme (NEP) (HAF NEP; *n* 17).

**Results:**

Kruskal–Wallis and Mann–Whitney *U* analyses found no significant between-group differences for any outcome, apart from perceived cooking competence. HAF NEP was associated with improved perceived cooking competence.

**Conclusions:**

Standard HAF was not associated with improved outcomes related to nutritional education. The HAF NEP group was associated with improved cooking competence only. The lack of significant findings in the intervention groups suggests that further research into HAF nutritional education is required.

Across the UK, an increasing number of households are facing food insecurity, with recent reports depicting that around 15 % of UK households are living with food insecurity^(1)^. To help attenuate food insecurity during school term-time, the Department for Education (DfE) provides means-tested free school meals (FSM) to children in England, ensuring that children from some of the most disadvantaged households receive a free, nutritious meal each school day that complies with School Food Standards^(2)^.

Whilst FSM provide a nutritional safety net for an increasing number of low-income families, recent research suggests that due to the strict means-tested criteria, approximately one in three children in England who are living in poverty are not eligible for FSM^(3)^. Moreover, FSM provision is unavailable to all families during school holiday periods, resulting in increased financial pressures and stretched budgets and, consequently, a type of food insecurity often termed ‘holiday hunger’^(4)^. Some local authorities use their Household Support Fund to provide families with FSM vouchers. However, whilst these vouchers may help to alleviate childhood food insecurity, they provide families with little support in terms of childcare and child-focused activities and may not be sufficient to alleviate household food insecurity.

To increase the availability of physical and cultural activities and childcare and to alleviate ‘holiday hunger’, in 2021, the DfE announced funding of around £220M per annum, for three financial years, across all higher-tier local authorities in England to support the Holiday Activities and Food (HAF) programme. HAF aims to provide children who are in receipt of FSM during term-time with free, nutritious meals that meet School Food Standards, as well as a range of enriching activities throughout the school holidays^(5)^. Notably, the North East of England is an area of high deprivation and has the highest rate of child poverty in England, with high levels of FSM and HAF attendance^(6)^, thus providing an appropriate context in which to study how HAF is delivered at the local authority level.

National HAF guidance is disseminated by the DfE to all local authorities and organisations delivering HAF to support the delivery and quality assurance of the programme. These guidelines list minimum quality standards, including the provision of at least one meal per day that complies with School Food Standards, a range of physical and enrichment activities, safeguarding of the children and families attending, and signposting and referring families to additional resources and support. Furthermore, the guidance requires the mandatory inclusion of nutritional education, which may be considered an extension of food education taught within the national curriculum at both primary and secondary level^(7)^. The inclusion of nutritional education within HAF is based on prior research findings that have shown practical and hands-on food education to consistently improve a range of skills-based and psychosocial outcomes, such as wellbeing, both for children and adults^(8–10)^. A range of programmes have been developed and implemented internationally based on experiential learning theory^(11)^, which suggests that attendees develop an initial interest through practical experiences with food and cooking before developing life-long food-related skills^(12,13)^. Further evidence demonstrating the benefits of nutritional education has been found across a number of studies that show attendance at practical cooking experiences, in both community and school settings, to be associated with improvements in cooking confidence, competence and food literacy, which may subsequently alter dietary habits and intake^(10,14–16)^. Moreover, youth cooking programmes have been found to improve health-related quality of life for children and adolescents who attend the sessions^(17)^ – an important consideration for HAF researchers, as previous research regarding children has recognised the association between lower socio-economic status and lower health-related quality of life^(14,18)^.

Despite the benefits associated with nutritional education in other contexts, to the authors’ knowledge, no quantitative studies have investigated whether this mandatory component of the HAF programme is associated with positive outcomes, such as children’s food-related behaviour, skills, knowledge and wellbeing. Whilst recent research has demonstrated perceived benefits of the nutritional education component of HAF^(19,20)^ including potential improvements in cooking confidence and competence, willingness to try new foods and understanding of food origins, no study has used a quantitative approach to explore the associations between nutritional education in the HAF programme and related outcomes.

As such, the aim of this study was to investigate whether HAF nutritional education was associated with changes in a range of outcomes for children, including (1) liking of trying new foods, (2) frequency of trying new foods, (3) perceived cooking competence, and (4) health-related quality of life.

## Methods

### Study context

Studies investigating nutritional education often omit a control group^(13)^; thus, including a control group was deemed important for this study. During summer 2022, one local authority in the North East of England piloted a 4-d nutritional education programme (NEP) as part of their HAF offer (HAF NEP). The local authority HAF lead supported the recruitment of primary schools (*n* 3) who delivered HAF over the summer. Schools supported in identifying self-selecting groups of children who did not attend HAF (No HAF), attended their school-based HAF club (HAF) or attended their school-based HAF club alongside an experiential NEP, which was delivered by a qualified food education teacher (HAF NEP). Further information about the HAF NEP intervention can be found below.

### Study design

A quantitative, quasi-experimental design was employed. Due to the quasi-experimental nature of the study, it was not possible to randomly assign children to the HAF and HAF NEP programmes as is customary in experimental designs. As a result, a quasi-experimental, mixed-factorial 3× 3 × 2 design was used. The first between-subjects factor was school, with three schools participating in the study. The second between-subjects factor was group, which had three levels: No HAF (children who did not attend any element of HAF), HAF (children who only attended a school-based HAF holiday club) and HAF NEP (children who attended a school-based HAF holiday club as well as an additional NEP). The within-subjects factor was time, with data collected within the week prior to the school summer holidays (time point 1) and data collected within the first week of the autumn term (time point 2). The dependent measures were (1) liking of trying new foods, (2) frequency of trying new foods, (3) cooking competence and (4) health-related quality of life.

### Participants

Ethical approval was granted by the Ethics Committee within the Faculty of Health and Life Sciences at Northumbria University (approval number: 49580). Three schools that had previously delivered HAF and were planning to deliver the HAF programme over summer 2022 were invited to take part in the study. The sampling frame consisted of all children in Year 4 and Year 5 attending one of the three schools participating in the study^(21)^.

After receiving consent from each school head teacher, and upon their request, school-based loco parentis opt-out consent was used. A non-probability purposive sampling strategy was used to invite children in Year 4 and Year 5, who attended one of the participating schools, to take part in the study. This age group was chosen because recent HAF evaluations show that 76 % of HAF attendees are of primary school age^(22)^ and because children of this age are capable of retrospective recall^(23,24)^ and capable of completing the CooC11^(25)^ and KIDSCREEN-10^(26)^ questionnaires. Overall, 254 children completed the measures at least once; 184 children completed the measures within the week prior to the school summer holidays (time point 1), and 207 children completed the measures within the first week of the autumn term (time point 2). The analysis for each outcome measure was conducted on the change scores from time point 1 to time point 2. Hence, only the data of children who completed the questionnaire at both time points were included in the subsequent data analysis. Eighty-five children who did not complete the measures at both time points were therefore omitted from subsequent data analysis, resulting in a final sample of 169 children, aged between 8 and 11 years old (mean age = 9·4 years, s d = 0·54). Based on HAF attendance over the school summer holidays, the self-selecting groups were No HAF (*n* 123), HAF (*n* 29) and HAF NEP (*n* 17) (see Table 2).

**Table 1.** Characteristics of the three schools in the school year 2021/2022

	School 1	School 2	School 3
Number of pupils on school roll	316	221	362
Percentage of pupils entitled to FSM (%)	67 %	48 %	47 %
Maximum number of days delivering HAF	12	16	8

FSM, free school meals; HAF, Holiday Activities and Food.

Source: Pupil population data downloaded from https://www.find-school-performance-data.service.gov.uk/ ^(21)^.

**Table 2.** Characteristics of children according to school and group

	School 1		School 2		School 3	
	Mean	sd	Mean	sd	Mean	sd
**No HAF**						
Number of children	37		33		53	
Mean age in years (sd)	9·4	0·50	9·4	0·56	9·5	0·58
Male	21		18		28	
Female	16		15		25	
**HAF**						
Number of children	1		8		20	
Mean age in years (sd)	9·0	0·00	9·4	0·52	9·3	0·57
Male	0		6		7	
Female	1		2		13	
**HAF NEP**						
Number of children	13		4		0	
Mean age in years (sd)	9·5	0·52	9·0	0·00	N/A	
Male	4		2		N/A	
Female	9		2		N/A	
**Total**	51		45		73	

HAF, Holiday Activities and Food; NEP, nutritional education programme.

### Materials

A paper-based battery of outcome measures was developed, which included self-reported demographic questions alongside all outcome measures. These data were collected from children as a group within their familiar classroom context.

## Liking and frequency of trying new foods (pictorial Likert scales)

Including pictures in scales can convey meaning and enhance inclusivity^(27)^. Hence, a 5-point pictorial Likert scale was used to collect data on children’s liking of trying new foods. Children were asked, ‘Do you like trying new foods?’, and responses ranged from ‘I really don’t like trying new foods’ to ‘I really like trying new foods’. For frequency of trying new foods, children were asked, ‘How often do you try new foods?’, with responses ranging from ‘Never’ to ‘Always’.

## Perceived cooking competence: CooC11

CooC11^(25)^ is an age-appropriate and validated measure suitable for use with children between 8 and 12 years old to measure perceived cooking competence. The scale includes eleven different cooking-related skills. Children view illustrations of characters completing each skill and then report whether they do the skill themselves (e.g. stirring/mixing ingredients). If the child responds with ‘yes’, they are shown two characters labelled A and B: one character completing the skill who is ‘really good’ and one character who is ‘not that good’. If the child self-reports that they do not do the skill, they receive a score of 0 for the skill and continue to questions about the next skill. However, if the child self-reports that they do the skill, they are asked to rate which character they are most like on a 5-point scale of ‘I am a lot like A’ to ‘I am a lot like B’. In line with the CooC11 guidance^(25)^, the presentation of whether the illustration is ‘really good’ or ‘not that good’ alternates, and therefore some scores are reverse coded, so higher scores indicate higher perceived cooking competence. A total score is computed by summing the scores from the eleven items (0–55).

## Health-related quality of life: KIDSCREEN-10

KIDSCREEN-10^(26)^ is a validated measure suitable for use with children aged 8–18 years and includes ten items that are rated using a 5-point scale to measure health-related quality of life. For this scale, children are asked to think about the previous week when responding to each item. As per the scoring instructions^(26)^, reverse scoring was used for item 3, ‘Have you felt sad?’, and item 4, ‘Have you felt lonely?’. A total score was derived by summing the item scores together, with a higher total score indicating a higher health-related quality of life and a lower score indicating a lower health-related quality of life.

### Procedure

**Figure 1.**
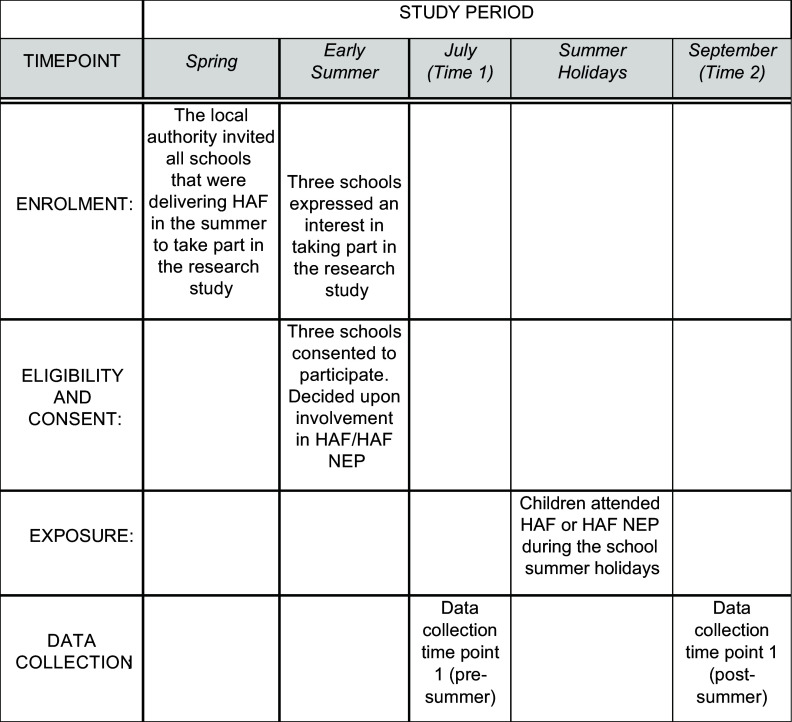
SPIRIT schedule of enrolment, eligibility and consent, exposure and data collection. HAF, Holiday Activities and Food; NEP, nutritional education programme.

A visual summary of the procedure is available in Figure 1.

One local authority in England, which was piloting a NEP as part of HAF during summer 2022, invited all schools delivering HAF to participate in the study. Three schools expressed interest and were sent detailed information about the study before consenting to take part. All three schools delivered a school-based HAF holiday club, and two schools included an option for children to attend a 4-d HAF practical NEP (HAF NEP) as part of the HAF programme. The delivery, location and the maximum number of children able to attend the additional HAF NEP sessions were decided by the local authority, based on practical elements such as funding, transport, timings and group sizes. All participating schools were located in areas of high deprivation (see Table 1), and all programmes were free of charge for the children to attend. Head teachers consented in loco-parentis. The researcher emailed letters to each school, which were then sent to parents/carers, containing detailed information about the study and how to withdraw their child from participation if required. The researcher then organised mutually convenient dates and times with each school for data collection to take place, on one day during the week prior to the school summer holidays and one day during the week following the school summer holidays.

On each day of data collection, children were given an information sheet about the study, which was also read aloud by the researcher. Although no children wished to withdraw from the study at this stage, all were reminded of their right to withdraw. To ensure anonymity and to link each individual child’s data across time points, all children were provided with a unique participant number; the researcher provided children with printed paper-based versions of the measures that were labelled with the corresponding participant number.

Each child completed the measures individually, but within a whole-class context. The researcher led the sessions by reading each individual question aloud to ensure enhanced clarity. School staff were on hand to support children if needed. To reduce fatigue, children were given a short, planned break during the completion of the measures. In total, the measures took approximately 50 min to complete. Children were offered a sticker as a thank you for participation in the study and were also provided with a written debrief following the final data collection. Debrief documents were emailed to each school, including debrief letters to be sent to parents/carers, which provided further details about the study and reminded parents/carers of their right to withdraw their child’s data. No children or data were withdrawn from the study during or after data collection.

### Nutritional education programme

Children were collected by minibus from their school-based HAF club on HAF NEP days and driven to a local secondary school. Children from school 1 and school 2 attended HAF NEP on different days but received identical sessions delivered by the same food education teacher; topics included food hygiene, safe storage, measuring ingredients, food preparation, knife skills, following recipes and cooking using a range of facilities. Children also made bread, chicken dishes, stir-fry and cupcakes. Each HAF NEP session lasted for 4 h/d, and children were offered to attend a total of 4 d of HAF NEP over 2 weeks in the school summer holiday (i.e. max 16 h). The children attended HAF NEP instead of their school-based HAF club on each respective day. At the end of each session, children were given a copy of the recipe they had followed. For the HAF group, providers delivered their standard practice following the DfE HAF guidance regarding nutritional education^(5)^.

### Treatment of data

**Figure 2.**
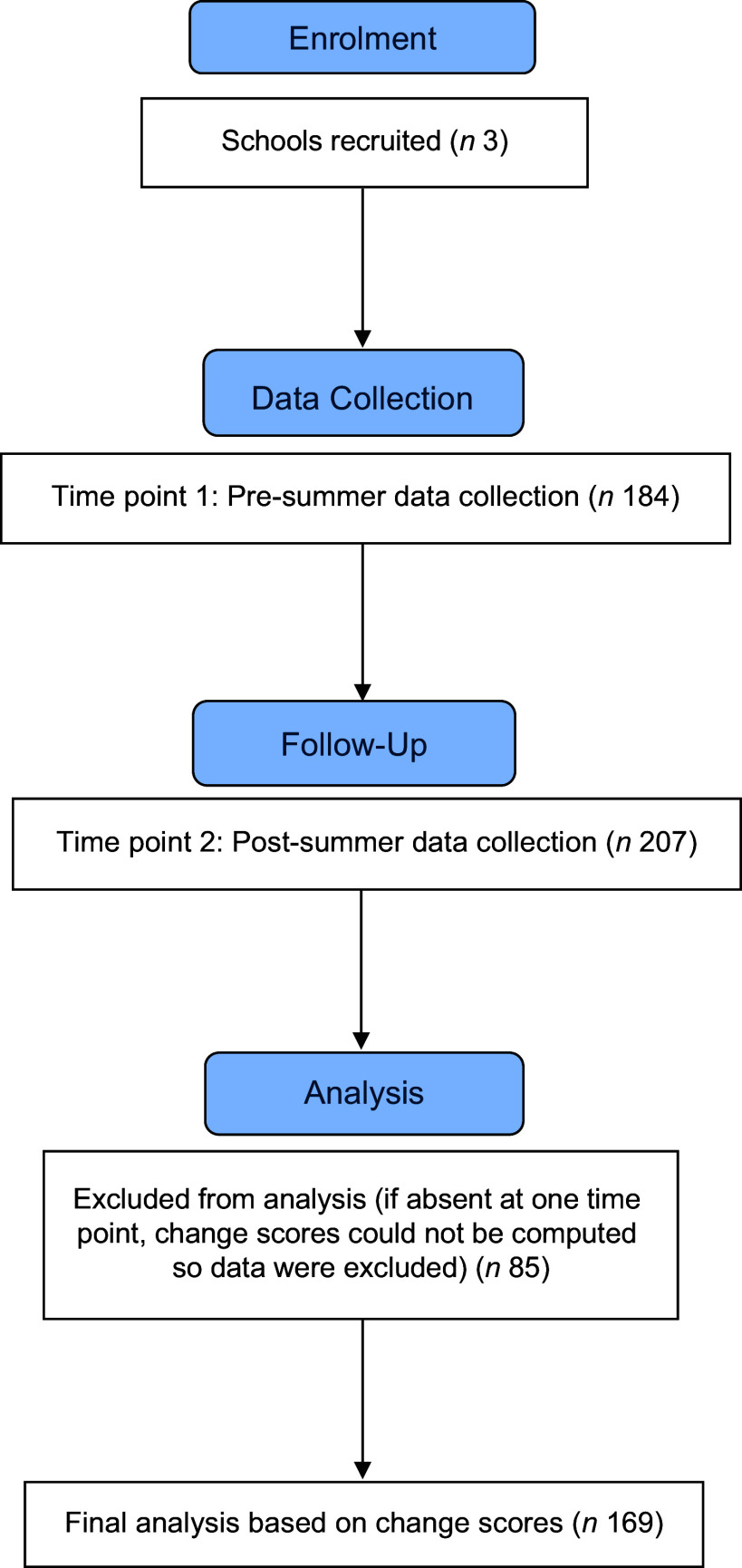
Participant flow diagram.

Data were collected using identical measures at both time points. At time point 1, data were collected from 184 children across all three schools. At time point 2, data were collected from 207 children across all three schools. The difference in the number of children participating in the study at the two time points was due to absenteeism. To compute change scores, data were removed from the data file for any child who did not complete the measures at both time points. Data were removed for 85 participants who only completed the measures once, resulting in a final sample size of 169 participants (see Figure 2 for a participant flow diagram).

Where appropriate, scoring instructions were followed to ensure accurate data scoring. To analyse change scores for each measure across time, total individual scores for each measure collected at time point 1 were subtracted from total individual scores for each measure collected at time point 2. As the data violated the homogeneity of variances assumption required for mixed ANOVA, change scores were computed and used for more conservative non-parametric analyses (Kruskal–Wallis followed by post hoc Mann–Whitney *U* tests). For any significant results, change scores were used for further ordinary least squares multiple linear regression analyses (controlling for age, gender, ethnicity, FSM status and school).

Finally, Spearman’s Rho^(28)^ was used to assess potential associations between (1) the total number of days that children in the HAF group attended their school-based HAF club and change in any significant outcome, and (2) the total number of days that children in the HAF NEP group attended HAF NEP (school-based HAF alongside the additional NEP) and change in any significant outcome.

## Results

Kruskal–Wallis analysis found no differences between schools for any outcome measure. Thus, data were collapsed across schools.

Kruskal–Wallis analysis was conducted to explore whether there were differences between any of the groups (No HAF, HAF, HAF NEP) in terms of (1) liking of trying new foods, (2) frequency of trying new foods, (3) cooking competence and (4) health-related quality of life (see Table 3 and Table 4). There were no significant between-group differences for any of the outcome measures, apart from perceived cooking competence. This analysis found a significant difference between groups for perceived cooking competence (*H*(2) = 7·573; *P* = 0·02). These associations are examined in greater detail using Mann–Whitney *U* tests for independent samples. Mann–Whitney *U* tests did not identify any statistically significant differences in perceived cooking competence scores between (1) children in the HAF group and the No HAF group (*U* = 1628(1), *P* = 0·47) or (2) children in the HAF group and HAF NEP group (*U* = 170·5(1), *P* = 0·08). There was, however, a statistically significant difference in change scores for perceived cooking competence between children in the HAF NEP group and children in the No HAF group, with significantly greater improvements for perceived cooking competence for the HAF NEP group (*U* = 615(1), *P* = 0·006). In particular, the median change in the cooking competence score for the HAF NEP group (median = 10) was 8 points higher than the median change in the cooking competence score for the No HAF group (median = 2).

**Table 3:** Median score (range) for each group according to time point

	No HAF				HAF				HAF NEP			
	Time 1		Time 2		Time 1		Time 2		Time 1		Time 2	
	Median	Range	Median	Range	Median	Range	Median	Range	Median	Range	Median	Range
**Outcome measure**												
Liking of trying new foods	3	1–5	3	2–5	4	2–5	4	1–5	4	2–5	4	2–5
Frequency of trying new foods	2	0–4	2	0–4	2	1–4	2	1–4	3	1–4	3	1–4
Perceived cooking competence	18	0–55	22	0–55	19	0–54	22	3–53	20	0–55	43	0–55
Health-related quality of life	39	20–49	40	24–49	37	23–48	38	29–49	42	29–49	44	25–48

HAF, Holiday Activities and Food; NEP, nutritional education programme.

**Table 4:** Kruskal–Wallis analyses for all outcome measures

Outcome measure	*H*	*df*	Sig (2-tailed)
Liking of trying new foods	0·61	2	0·74
Frequency of trying new foods	0·01	2	0·99
Cooking competence	7·57*	2	0·02*
Health-related quality of life	0·78	2	0·68

* Significant at the 0·05 level (2-tailed).

Ordinary least squares regression was also used to assess the association between cooking competence and HAF attendance whilst controlling for age, gender, ethnicity, FSM status and school (Appendix A). Notably, controlling for school accounted for the clustering of pupils within schools and adjusted for potential within-school dependence. Together, the independent variables were statistically significant (*F*(8160) = 2·413, *P* = 0·017) with an adjusted *R* ^2^ of .063 (showing that the model explains 6·35 % of the variance in perceived cooking competence). In that model, HAF NEP was the only variable associated with perceived cooking competence (*B* = 9·75, *t* = 3·35, *P* = 0·001). In this sample, perceived cooking competence scores were 9·75 points higher amongst the HAF NEP group than the No HAF group. This finding is consistent with the result of the Kruskal–Wallis analysis.

A final analysis used Spearman’s Rho to explore the potential association between perceived cooking competence and the number of days children attended HAF for (1) children in the school-based HAF programme and (2) children in the NEP-based HAF programme. No statistically significant associations were apparent for the total number of days that children in the HAF group attended school-based HAF and change in perceived cooking competence (*r*(27) = 0·019, *n* 29, *P* = 0·924). Likewise, no significant associations were observed between the total number of days that children in the HAF NEP group attended HAF NEP (school-based HAF alongside the NEP) and change in perceived cooking competence (*r(*27) = –.203, *n* 29, *P* = 0·435).

## Discussion

The aim of this study was to investigate whether nutritional education, within the HAF programme, was associated with improved outcomes in (1) liking of trying new foods, (2) frequency of trying new foods, (3) perceived cooking competence, and (4) health-related quality of life. Overall, standard HAF was not associated with improvements in any of these outcome measures, and HAF NEP was not associated with improvements in liking of trying new foods, frequency of trying new foods, or health-related quality of life. However, HAF NEP was associated with improved cooking competence, which is notable as the HAF NEP sessions were focused on providing practical food preparation and cooking opportunities.

Although nutritional education is a mandatory component of the DfE’s HAF programme, the HAF group was not significantly associated with changes in any outcome for children who attended. This contradicts prior studies that have adopted a qualitative approach to report the perceptions of HAF leads^(19,20)^. Furthermore, these findings do not lend support to other studies regarding nutritional education interventions in non-HAF contexts, where attendance is often associated with improvements in cooking competence and willingness to try new foods^(8,15–17)^. Typically, however, nutritional education interventions across non-HAF contexts exclusively deliver food-related content as the sole focus of their sessions. Previous research has highlighted that interventions that provide more than 6 h of cooking activities are often more effective in improving cooking confidence and vegetable intake, acknowledging a dose–response that longer, more sustained interventions are more likely to improve related outcomes^(29)^. By contrast, however, within the context of HAF provision, nutritional education is only one component of the wider HAF programme, where providers are also required to deliver a range of enrichment and physical activities^(5)^. Hence, there may be several barriers in terms of implementation and delivery of nutritional education within HAF when considering the current operational structure of the programme, which have been recognised in previous research with HAF leads and providers^(19,20)^. First, staff and volunteers within HAF settings often experience challenges in finding enough time during the day to deliver nutritional education alongside other required enrichment activities (e.g. physical activities, day trips, arts)^(20)^. A further challenge relates to the expertise of delivery staff who may not be confident in delivering nutritional education^(20)^. Many local authorities utilise shared resources that are frequently used in schools (e.g. Eatwell Guide), but it is not clear whether such resources scaffold and build upon existing knowledge states or whether content is age-appropriate for the diverse range of young people attending HAF provision. Providers also acknowledge limitations with the kitchen facilities and amenities that HAF settings often hold, which can limit the type and quality of nutritional education that can be offered^(19, 20)^. Thus, time, resources and staff expertise may limit the experiential learning activities offered to children and young people.

The only group to show an improvement in cooking competence was the HAF NEP group that provided children with access to four structured, intensive and hands-on cooking sessions, and this was led by a food technology teacher. These findings are supported by experiential learning theory^(11)^, which recognises a practical approach to learning as valuable in creating an initial interest in the subject matter, which often leads to continued skill development^(15)^. HAF typically involves children from low-income households, who can have limited experience of cooking and food preparation due to the unaffordable risk of food waste and spoilage^(30)^. HAF NEP provided a novel opportunity for children to engage in multiple, hands-on cooking experiences and to develop an interest in cooking and food preparation skill development and thus was associated with improved perceived cooking competence.

In addition, prior research has shown that food-related interventions are often more beneficial when led by a culinary professional, as was the case in the HAF NEP group^(8,15,16)^. However, the outsourcing of skilled professionals to deliver HAF sessions is often limited by funding^(20,31,32)^; thus, implementing similar sessions in HAF clubs may prove challenging.

However, HAF NEP was not associated with improvements in liking and frequency of trying new foods or health-related quality of life. Given that HAF leads in prior qualitative work perceived improvements in these outcomes related to the nutritional education component of HAF^(19,20)^, and studies within non-HAF contexts have often found NEP to improve children’s willingness to try new foods, food literacy and a range of psychosocial impacts^(10,14,33)^, this finding was surprising. One explanation relates to research which has shown that children typically require 10–15 repeated exposures to new foods before any significant food-related behaviour change is notable^(34–36)^. Whilst the HAF NEP sessions afforded children repeated opportunities to develop food-related skills to improve their perceived cooking competence, the focus of each HAF NEP session was tailored to a new food type each day, thus potentially limiting the amount of repeated tasting experiences and repeated exposure to foods that are required to drive changes in liking and the frequency of trying new foods. Whilst it is recognised that repeated food exposure in sustained food education sessions is key to improving these outcomes and overall dietary intakes^(37)^, the current funding and operational structure of HAF, where providers are required to deliver a broad spectrum of enriching and physical activities within a short daily delivery model and with tight budgetary constraints and timeframes, may limit the implementation and delivery of such repeated food exposure activities and opportunities^(5,20)^.

In addition, no significant improvements were found for health-related quality of life, which is a multi-dimensional term concerning wellbeing; comprising physical, emotional and social aspects. Brennan *et al*.^(14)^ similarly used the KIDSCREEN-10^(26)^ to measure health-related quality of life and found no significant improvement following attendance at a practical NEP. However, Saxe-Custack *et al*.^(17)^ instead measured health-related quality of life through the Paediatric Quality of Life Inventory^(38)^ and found significant improvements in all measured aspects for children, following participation in a cooking programme similar to HAF NEP. Whilst both measures are valid and age-appropriate, the Paediatric Quality of Life Inventory separates health-related quality of life into four sub-components: physical functioning, emotional functioning, social functioning and school functioning. Future research regarding health-related quality of life and the HAF programme should therefore consider utilising this measure, particularly as attendance at holiday provision is related to emotional wellbeing and socialisation^(31,39,40)^.

Lavelle^(13)^ highlighted that many studies investigating nutritional education interventions fail to incorporate a control group for comparison. Including a control group within the current study served as a baseline to determine whether outcomes changed without access to HAF or HAF NEP. This was particularly important as although the No HAF, HAF and HAF NEP groups were self-selecting and hence, causality could not be inferred, between-group comparisons could be made in relation to each of the outcome measures.

In addition, it is important to note that data were collected from only one local authority in the North East of England and from only three schools within this local authority that were willing to take part. As HAF is both an asset-based and place-based programme, and prior research notes variation in how nutritional education is delivered both across and within local authorities^(19,20)^, future research is warranted, which considers a national spread of local authorities and schools.

Nevertheless, the findings within this study further this under-researched component of HAF. Indeed, this is the first study to use a quantitative approach to explore nutritional education within the HAF programme. HAF NEP was found to be the only group associated with improved perceived cooking competence, which suggests the importance of practical and experiential learning within HAF. However, performance in the HAF group, which reflects typical HAF provision, was not associated with any improved outcomes. Together with other wider international evidence on the importance of practical cooking interventions for children^(29, 31, 37)^, these findings across the two intervention groups, compared to the control, suggest that the DfE should promote the inclusion of hands-on, expert-led, experiential nutritional education within HAF.

## Supporting information

Hope et al. supplementary material Hope et al. supplementary material

## Supplementary material

To view supplementary material for this article, please visit https://doi.org/10.1017/S1368980026102225.
