# Supplementary material for: A quasi-experimental study to explore the association between nutritional education within the Holiday Activities and Food programme and related outcomes for children
Source: Public Health Nutr. 2026 Feb 27;29(1):e59. doi: 10.1017/S1368980026102225 (PMC13112297; doi:10.1017/S1368980026102225)
Supplement: Hope et al. supplementary material [file S1368980026102225sup001.docx]

**Appendix A. OLS Estimates of the Effect of Ethnicity, Gender, Age, FSM, School and Nutritional Education on Cooking Competence**

| Variable | Coef. |  |
| --- | --- | --- |
|  | (Std. Error) |  |
|  |  |  |
| *Child’s Ethnicity* (1=*White*, 0=*Not White*) | -0.46 |  |
|  | (1.98) |  |
| *Child’s Gender* (1=*Male*, 0=*Female*) | -2.66 |  |
|  | (1.68) |  |
| *Child Receives FSM* (1=*Yes*, 0=*No*) | 2.07 |  |
|  | (1.70) |  |
| *Child’s Age* (in years) | -0.65 |  |
|  | (1.58) |  |
| Schools (vs. *School 3*) |  |  |
|  |  |  |
| *School 1* | -1.96 |  |
|  | (2.21) |  |
| *School 2* | 1.51 |  |
|  | (2.09) |  |
| Programme (vs. *No Programme*) |  |  |
|  |  |  |
| *HAF NEP* (*Nutritional Education*) | 9.75 | * |
|  | (2.91) |  |
| *HAF Only* | -1.97 |  |
|  | (2.26) |  |
| Constant | 10.4 |  |
|  | (15.5) |  |
|  |  |  |
| n | 169 |  |
| Adj. R-Square | 0.063 |  |
| * Significant at the 0.05 level (2-tailed) |  | . |
